# Emergence Patterns of Rare Arable Plants and Conservation Implications

**DOI:** 10.3390/plants9030309

**Published:** 2020-03-01

**Authors:** Joel Torra, Frank Forcella, Jordi Recasens, Aritz Royo-Esnal

**Affiliations:** 1 Department of Hortofruticultura, Botànica i Jardineria, Agrotecnio, Universitat de Lleida. Alcalde Rovira Roure 191, 25198 Lleida, Spain; jordi.recasens@udl.cat; 2 Department of Agronomy and Plant Genetics, College of Food, Agricultural and Natural Resources Sciences (CFANS), University of Minnesota, St Paul, MN 55108, USA; force002@umn.edu

**Keywords:** burial depth, Calinski–Harabasz criterion, clustering, K-means, multivariate analyses, no-tillage, Principal Component Analysis (PCA), soil disturbance, tillage

## Abstract

Knowledge on the emergence patterns of rare arable plants (RAP) is essential to design their conservation in Europe. This study hypothesizes that is possible to find functional groups with similar emergence patterns within RAP with the aim of establishing management strategies. Seeds of 30 different species were collected from Spanish arable fields and sown under two tillage treatments: (a) 1 cm depth without soil disturbance to simulate no-till, and (b) 1–10 cm depth with soil disturbance every autumn to simulate tillage to 10 cm depth. Two trials were established; the first trial being maintained for three seasons and the second for two seasons. Relative emergence in autumn, winter and spring was calculated each season. Afterwards, multivariate analysis was performed by K-means clustering and Principal Component Analysis to find groups of RAP species with similar emergence patterns. Four RAP groups were defined, and each was based on its main emergence season: autumn, winter, spring, or autumn-winter. Tillage treatment and the year of sowing had little effect on emergence patterns, which were mostly dependent on environmental factors, particularly temperature and rainfall. Therefore, conservation strategies could be designed for each of these RAP functional groups based on emergence patterns, rather than on a species-by-species basis.

## 1. Introduction

The ecosystem in Europe most influenced by human activity is arable land [1]. Since the advent of modern agriculture, arable plant diversity has suffered a steep decline across the continent (reviewed in Albrecht et al. [2] and Richner et al. [3]). Probably, there is no other vegetation type in Europe in which populations have been so strongly reduced in qualitative and quantitative ways [1]. A particular set of specialist plants typically found in arable land, called segetal species, have suffered the most drastic population declines in recent years, to the point of becoming rare arable plants (RAP) or even locally extinct in many countries [4,5]. Therefore, an urgent need exists in Europe to prevent the strong decline in plant diversity in arable agro-ecosystems [6]. Particularly needed is the development of conservation and management strategies focused on the threatened RAP species [7].

Several studies consider how RAP could be conserved, although data on this subject is still very scarce and insufficiently documented [2] especially in the Mediterranean countries [8]. Conservation headlands, field flora reserves, uncropped cultivated margins and wildflower strips included within the Agro-environmental schemes (AES) of the European Union are the most widespread conservation measures [2]. However, most AES projects fail often after an initially successful phase mainly due to complicated and insecure funding [9] or expansion of highly competitive weeds [10]. Other favorable management practices to successfully establish RAP species are reduced crop densities, lower nitrogen fertilization, avoidance of herbicides, and medium sowing densities of the rare species [11]. However, there is still a lack of knowledge on best management strategies to promote establishment and abundance of RAP under real farm conditions over the long term.

The timing of seedling emergence is crucial for fitness of annual plants [12], which is why tillage is one of the most important anthropogenic drivers of weed communities [13]. Arable flora, such as RAP, is highly adapted to regular soil disturbances for thousands of years [14]. For this reason, the timing of tillage and sowing are other key factors affecting the success of arable plant establishment [15]. As most threatened arable plant species are autumn-winter annuals [16], a sowing date in autumn could increase the sustainable establishment of RAP species [15]. However, most studies deal only with a few species or emergence data are not provided to support the hypothesis. The same applies to the type of soil tillage management, as almost no literature provides data on how tillage can affect RAP populations. Recent research on the effects of tillage type (no-till versus till) on a large set of RAP showed that recurrent annual tillage could be important to maintain and replenish seed banks of these species for the mid to long-term, particularly for those with secondary dormancy processes and high seed bank persistence [5].

It is no doubt that conservation measures need to be established that integrate RAP species preservation into regular farming activities. However, RAP are almost absent from many areas [17,18] and, therefore, their reintroduction provides another measure to support plant diversity. Sowing dates and soil tillage management for RAP species may play important roles for successful reintroduction, in addition to suitable site and management conditions [10,19].

Importantly, because this approach has the risk of establishing pernicious weeds, reintroduction of RAP will only gain acceptance among farmers if yield losses due to weed competition are low [20]. Delayed sowing can reduce competition by early emerging and highly competitive weeds, mostly grass species [21]. Additionally, RAP usually have relatively poor competitive abilities with arable crops [22]. Moreover, there is consistently strong evidence that maintenance of high plant diversity is not a detriment to crop yields [23]. However, there is little published information on the effects of tillage or sowing dates for RAP and their related RAP-crop interactions [20]. Moreover, there are almost no available data on RAP emergence patterns, which would allow assessment of the effects of changing sowing times so that these species can be favored compared to common problematic weeds.

Plant species can be grouped into functional groups using their traits based on ecological strategies [24]. Identifying functional response groups of species with a similar response to a particular environmental factor, is a long-standing practice in weed control that is used to optimize weeding [13], although it could be also used for other objectives, such as preservation. Therefore, defining functional groups of RAP could be suitable for predicting the impact of management practices and environmental factors on this set of threatened species in arable fields and agricultural landscapes for restoration and conservation purposes. There are 193 arable plant species either on the national Red Data Lists or considered threatened in at least three of 29 European countries, and of those, 48 are critically endangered (16 are within this study) [6]. Though species-specific recommendations would be appropriate [5,7,25], such a research commitment is hardly attainable considering the numbers of RAP. Therefore, establishing functional groups to manage RAP would be highly desirable for practical recommendations to deliver maximum benefits for the widest range of species potentially present at a site.

To develop methods for restoring and increasing plant agro-biodiversity, with focus on RAP, this study aimed to analyze the emergence patterns of a large set of species. This research attempts to provide new data and summarize scarce available information to identify sowing dates and soil tillage regimes to optimize conservation strategies and successful establishment of 30 RAP species [17,26]. Thus, emergence patterns of these species were studied during three consecutive seasons in a cereal field in Spain under two distinct tillage regimes (no-till versus till). Afterwards, relative emergences in autumn, winter and spring of each season were estimated for each species. With these data, a multivariate analysis approach was performed to find groups of RAP with similar emergence patterns. This methodology is aimed at providing conservation strategies that can be applied to RAP of similar emergence patterns as alternatives to inefficient tactics based on species-specific recommendations.

## 2. Results

The three seasons were different in terms of rainfall and temperature (Table 1). Mean air temperatures were much higher the first season (2012/2013) from autumn to spring every month (at least two-fold) compared to the two following seasons. The coldest autumn was the second season (2013/2014), while the coldest winter was in the third (2014/2015). In contrast, the wettest autumn was in the third season (particularly in September and November), while the driest was the second (September and October). Winter was wettest in 2013/2014; while spring was very dry in 2014/2015 (Table 1).

**Figure 1:**
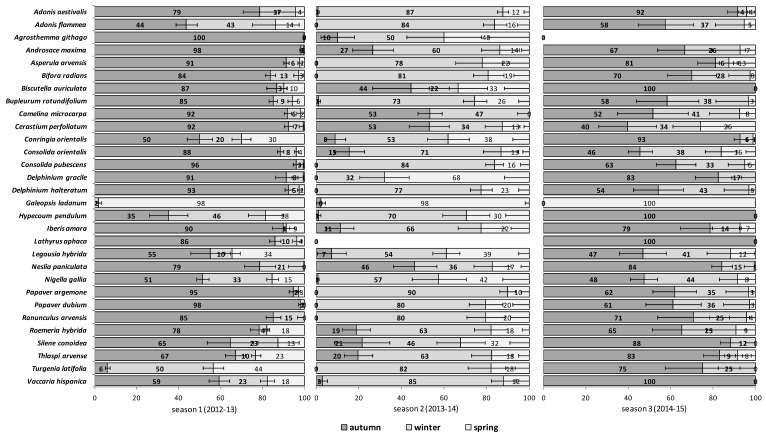
Relative Emergence patterns of 30 rare arable plants (RAP) in autumn (October through December), winter (January through February), and spring (March through April) during three consecutive seasons for seeds sown in 2012 (trial S1) in an arable field trial in Spain. Bars represent standard error. Two burial conditions are shown averaged: 1 cm burial depth without soil disturbance and 1–10 cm depth with annual autumnal soil disturbance. See Appendix A for relative emergence for each burial condition.

**Figure 2:**
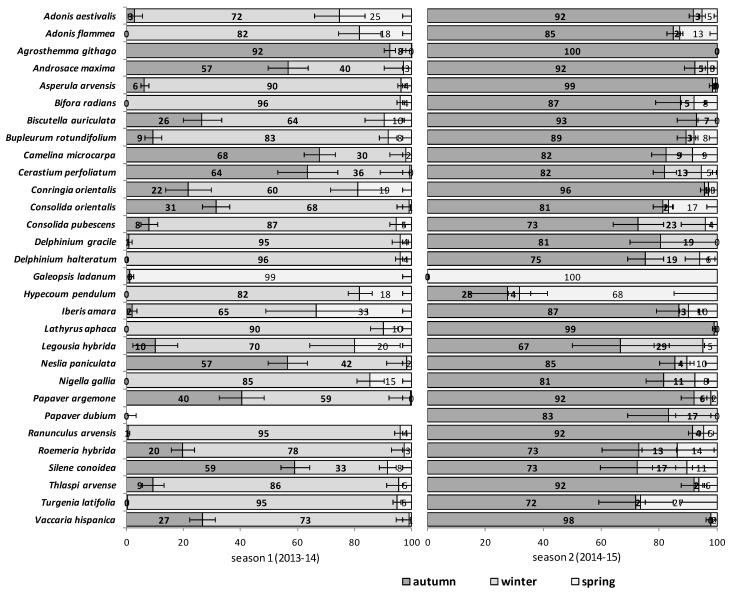
Relative Emergence patterns of 30 RAP in autumn (October through December), winter (January through February), and spring (March through April) during two consecutive seasons for seeds sown in 2013 (trial S2) in an arable field trial in Spain. Bars represent standard error. Two burial conditions are shown averaged: 1 cm burial depth without soil disturbance and 1–10 cm depth with annual autumnal soil disturbance. See Appendix A for relative emergence for each burial condition.

The emergence patterns in autumn, winter and spring, irrespective of the burial conditions (no-till at 1 cm depth vs. till at 1–10 cm depth), are shown in Figure 1 for the first trial (S1) and in Figure 2 for the second trial (S2). Patterns differed between trials. The first season in S1 (2012/2013) 25 of 30 RAP had at least 50% of their emergence in autumn, while the first season in S2 (2013/2014), this pattern existed only for 6 species: *Agrostemma githago*, *Androsace maxima*, *Camelina microcarpa*, *Cerastium perfoliatum*, *Neslia paniculata* and *Silene conoidea*. Results were reversed in the second season; in S1 (2013/2014) emergence was concentrated in winter and secondarily in spring (excepting *Biscutella auriculata*, *C. microcarpa*, *C. perfoliatum* and *N. paniculata* with important emergence in autumn), whereas in S2 (2014/2015) most species (28 out of 30) had at least 67% of emergence in autumn. The only exceptions were *Hypecoum pendulum* and *Galeopsis ladanum*. The latter species was the only one with a restricted spring emergence behavior since at least 98% of emergence occurred in that period irrespective of trial (S1 and S2) and season (Figure 1 and Figure 2).

**Figure A1:**
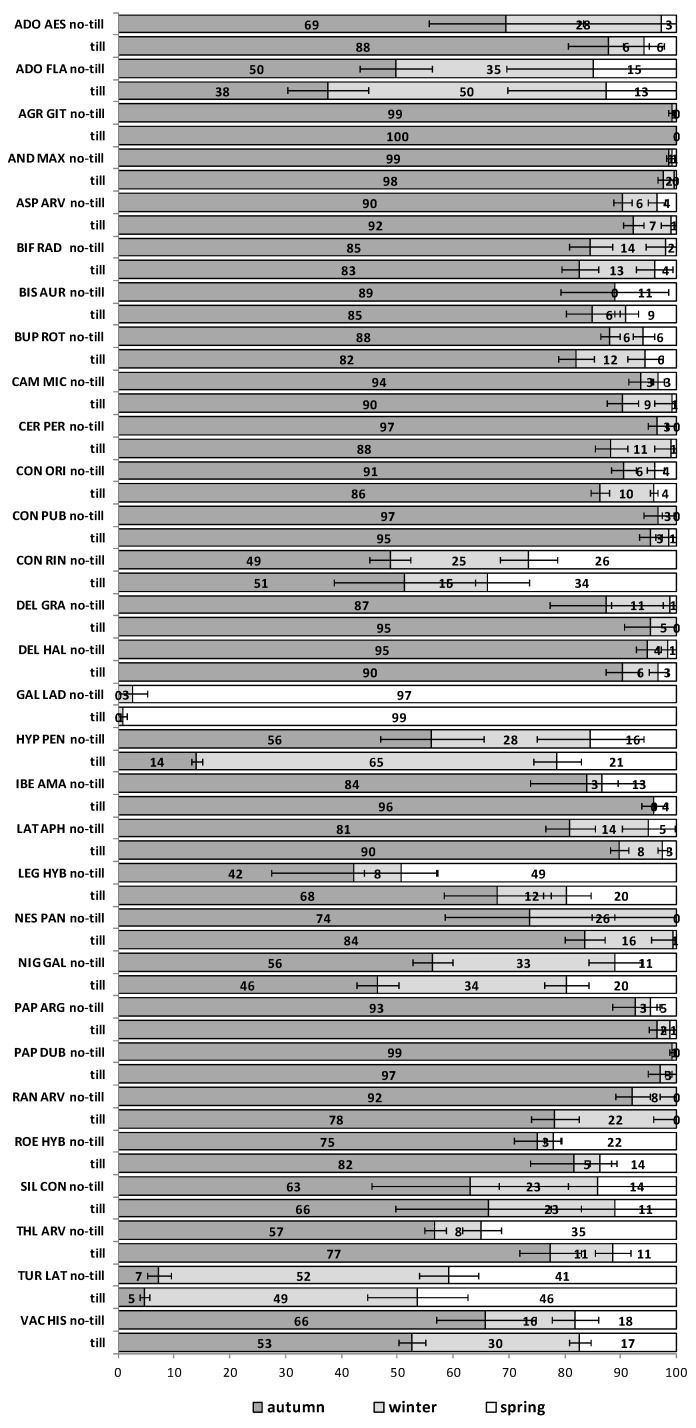
Relative Emergence patterns of 30 RAPs in autumn, winter, and spring during the first season (2012/2013) for seeds sown in 2012 (S1) in an arable field trial in Spain. Bars represent standard error. Two burial conditions are shown: 1 cm burial depth without soil disturbance (no-till) and 1–10 cm depth with annual autumnal soil disturbance (till).

**Figure A2:**
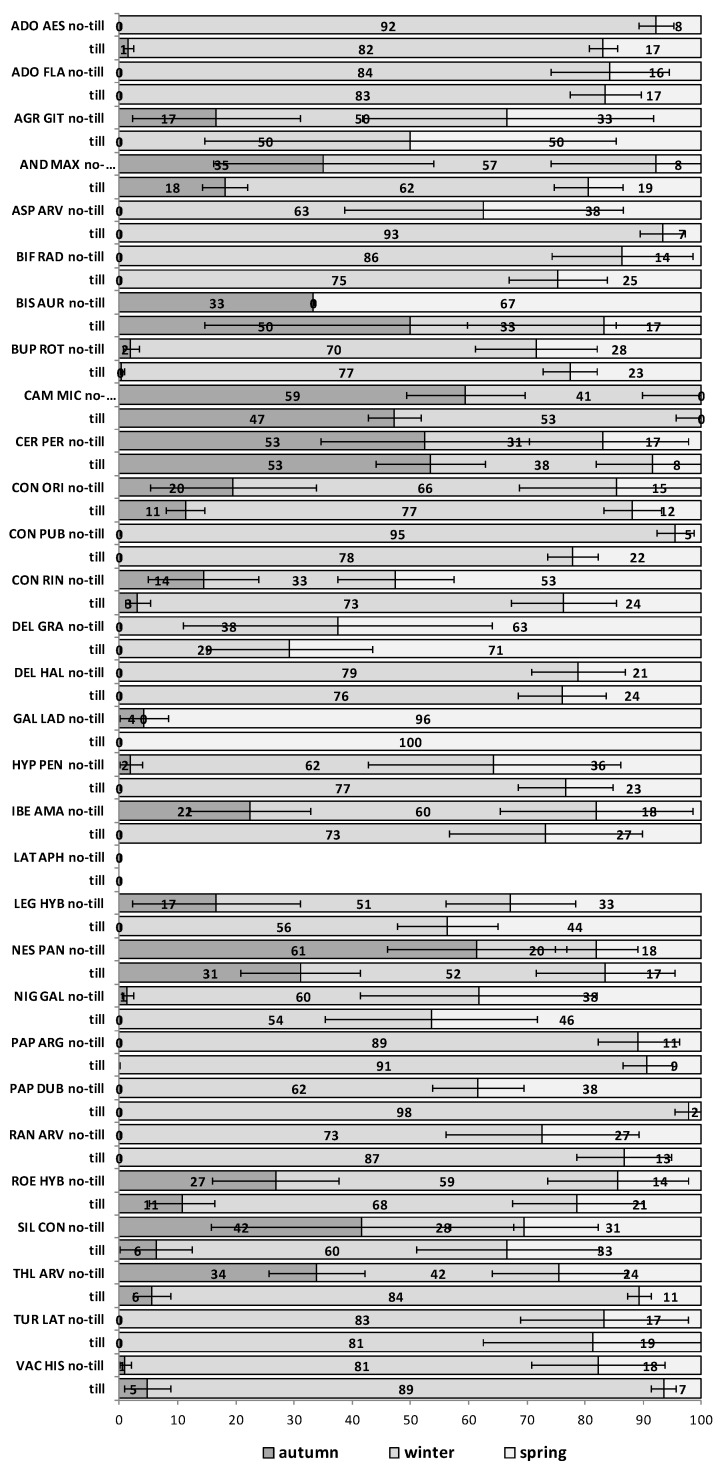
Relative Emergence patterns of 30 RAPs in autumn, winter, and spring during the second season (2013/2014) for seeds sown in 2012 (S1) in an arable field trial in Spain. Bars represent standard error. Two burial conditions are shown: 1 cm burial depth without soil disturbance (no-till) and 1–10 cm depth with annual autumnal soil disturbance (till).

**Figure A3:**
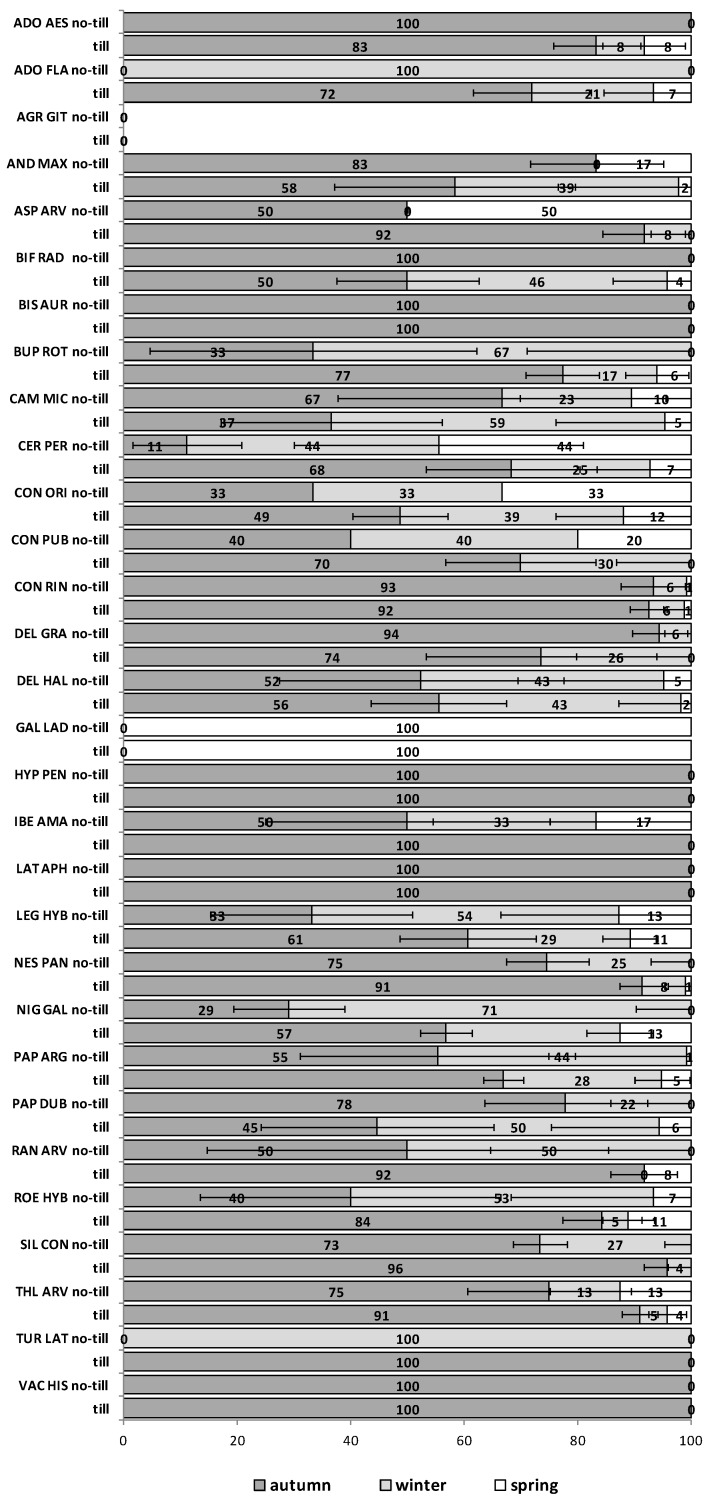
Relative Emergence patterns of 30 RAPs in autumn, winter, and spring during the third season (2014/2015) for seeds sown in 2012 (S1) in an arable field trial in Spain. Bars represent standard error. Two burial conditions are shown: 1 cm burial depth without soil disturbance (no-till) and 1–10 cm depth with annual autumnal soil disturbance (till).

**Figure A4:**
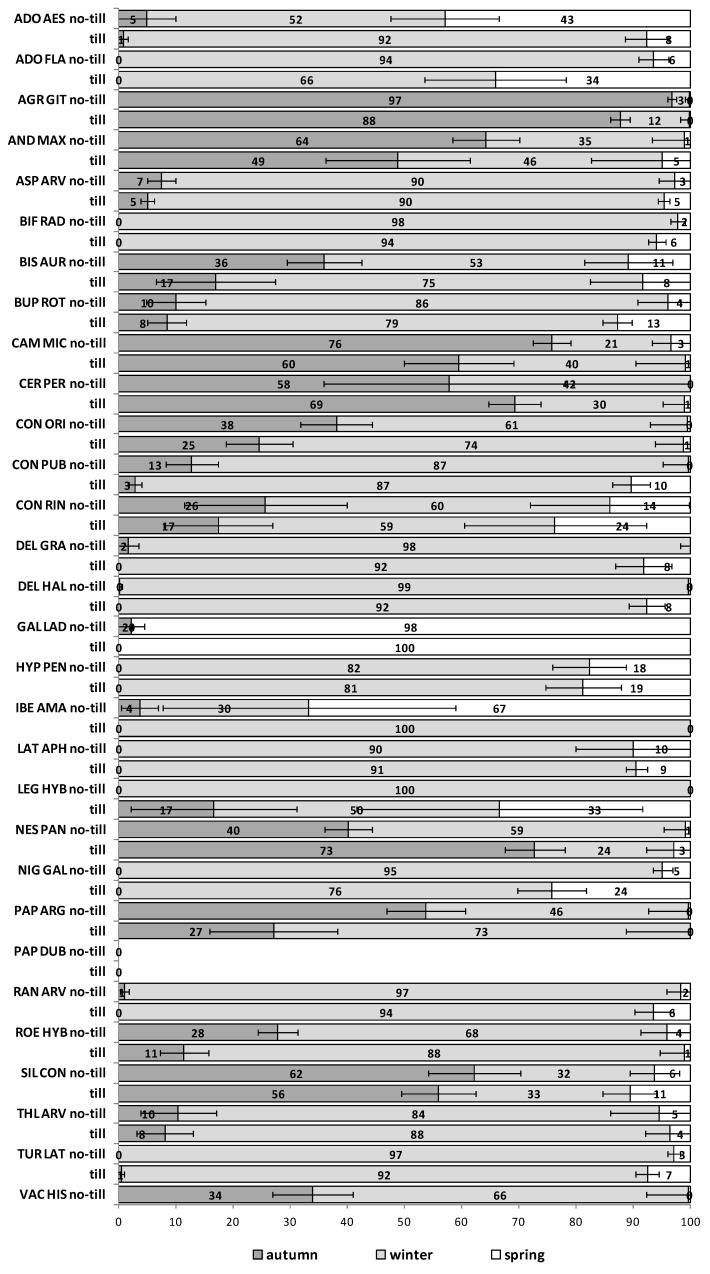
Relative Emergence patterns of 30 RAPs in autumn, winter, and spring during the first season (2013/2014) for seeds sown in 2013 (S2) in an arable field trial in Spain. Bars represent standard error. Two burial conditions are shown: 1 cm burial depth without soil disturbance (no-till) and 1–10 cm depth with annual autumnal soil disturbance (till).

**Figure A5:**
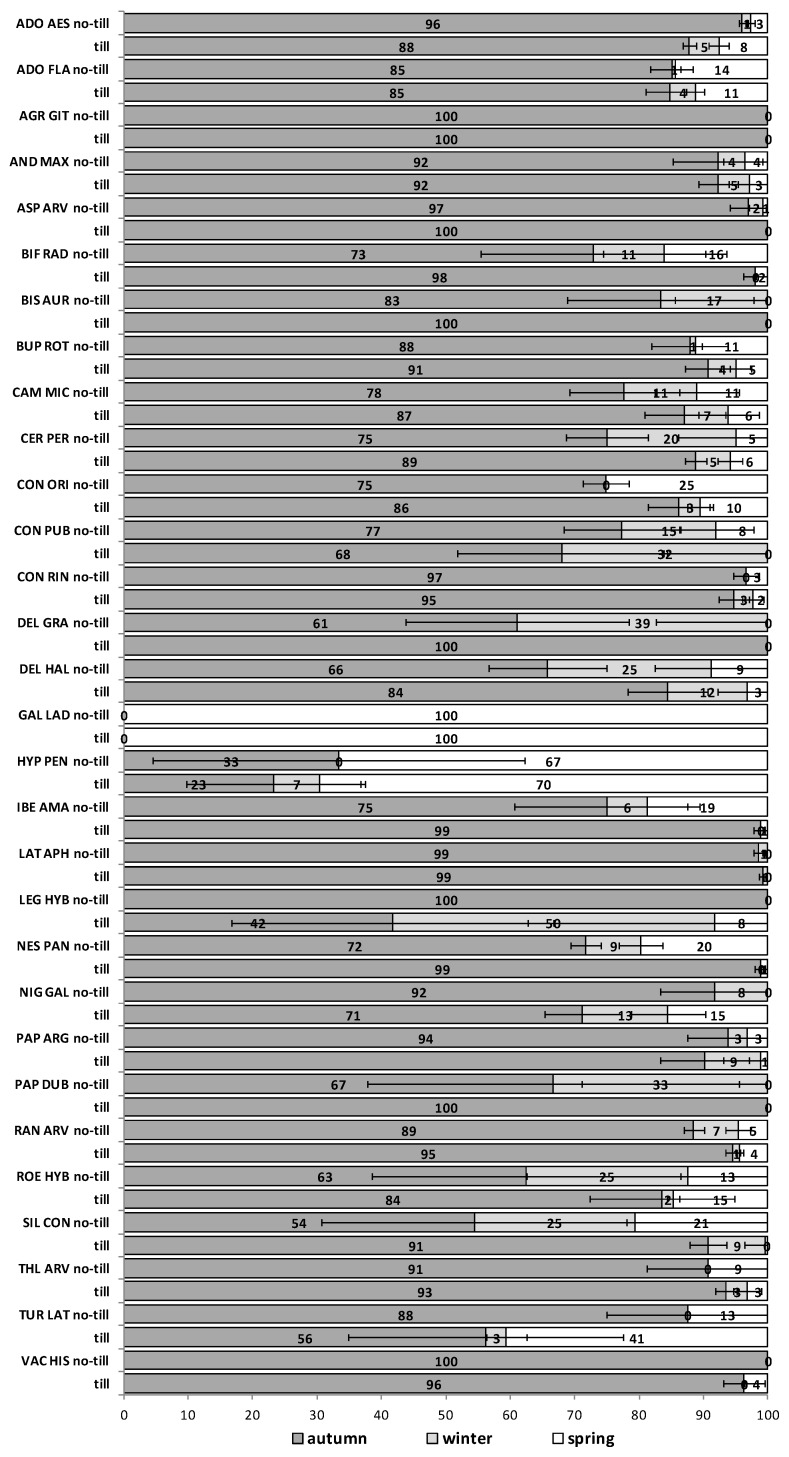
Relative Emergence patterns of 30 RAPs in autumn, winter, and spring during the second season (2014/2015) for seeds sown in 2013 (S2) in an arable field trial in Spain. Bars represent standard error. Two burial conditions are shown: 1 cm burial depth without soil disturbance (no-till) and 1–10 cm depth with annual autumnal soil disturbance (till).

The soil disturbance regimes, no-till vs. till, in general, did not influence appreciably the emergence patterns among the 30 RAP or between trials S1 and S2 across seasons (Figure A1, Figure A2, Figure A3, Figure A4 and Figure A5). In some cases, soil disturbance could delay, advance or not have any particular effect on the emergence of the 30 RAP depending on the species, trial and season. For example, in the first season in trial S1 emergence was delayed in seven species and advanced in another seven, in the second season it was delayed in 13 species and only advanced in three, while the third season emergence was delayed in six species and advanced in 15 (Figure A1, Figure A2 and Figure A3). Similarly, in the first season in trial S2 emergence was delayed by soil disturbance in 12 species and advanced in five, while in the second season it was delayed only in four and advanced in 12 species (Figure A4 and Figure A5).

### Multivariate Analysis

**Figure 3:**
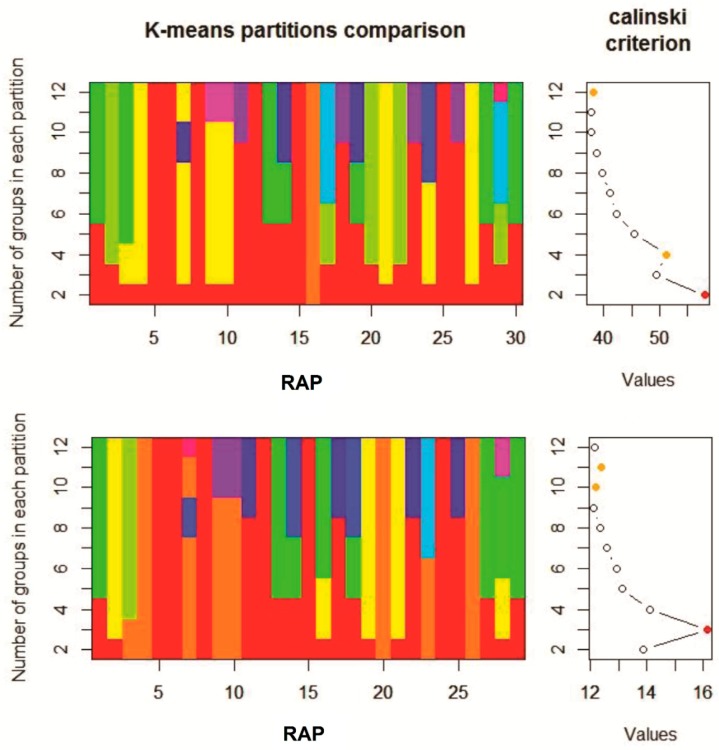
Results of the two K-means clustering applied with the Calinski–Harabasz criterion (CHC). The CHC indicated that the 30 RAP could be clustered into two groups (1 species in one, 29 in the second) in a first analysis (top chart), also marked as a red point on the linear plot of CHC values above on the right. Second K-means clustering with 29 species, shown in the bottom chart). The CHC indicated that the 29 RAP could be clustered into three groups, (red point in the linear plot of CHC values below, equivalent to the first orange point in CHC plot shown above). Colors within each of the columns of the figures on the left (30 above and 29 below) are groups of RAP in the K-means calculations.

The K-means clustering and the Calinski-Harabasz criterion (CHC) values identified only two clusters in a first analysis (Figure 3, top chart). Since only one species (*G. ladanum*) was ascribed to the first group and the rest (29 species) in the second group, and that the linear CHC plot indicated also the secondary presence of four significant groups (Figure 3, top chart, orange dot), a second K-means analysis was performed removing *G. ladanum* from the data set. In this second clustering, three significant groups were identified according to the CHC values (Figure 3, bottom chart, red dot). Species composition was the same in these three groups in the first and second cluster analysis. Considering both K-means cluster analysis altogether four distinct groups of RAP were identified according to their emergence patterns during the emergence season, as shown in Table 2.

**Figure 4:**
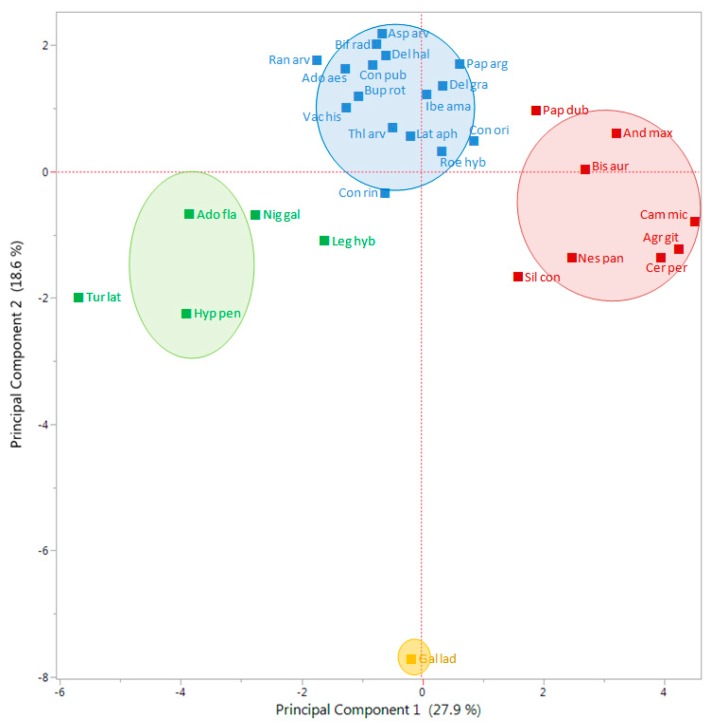
Biplot representation of K-means clustering using the first two PC of the PCA for 30 RAP species (three first letters of the genus and species) according to their emergence patterns in two trials (S1 and S2) during three consecutive seasons. There were two burial conditions considered in the analysis: 1 cm burial depth without soil disturbance and 1–10 cm depth with annual autumnal soil disturbance. Percentages in parenthesis represent the variability explained by each PC. Circles, ellipses and names with the same colors include the species within each of the groups derived from a K-means clustering of the ordination scores. The full species names are given in Table 2.

The first Principal Component (PC) of the Principal Component Analysis (PCA) explained 27.9% of the total variation, while the second PC explained 18.6%, which overall accounted for nearly 46.5% of the total variation (Figure 4). The PCA divided all studied species in two main groups, in one leaving *G. ladanum* isolated, in the other the rest of the 29 species (Figure 4). In the second group, an important number of species were close to the center coordinates 0.0 (i.e., *Consolida* or *Delphinium* species), while some species were located in the right part, such as *A. githago*, *A. maxima*, *B. auriculata*, *C. microcarpa*, *C. perfoliatum*, and *N. paniculata*, or some others on the left part of the PCA biplot (*Adonis flammea*, *H. pendulum*, *Nigella gallica*, and *Turgenia latifolia*).

**Figure 5:**
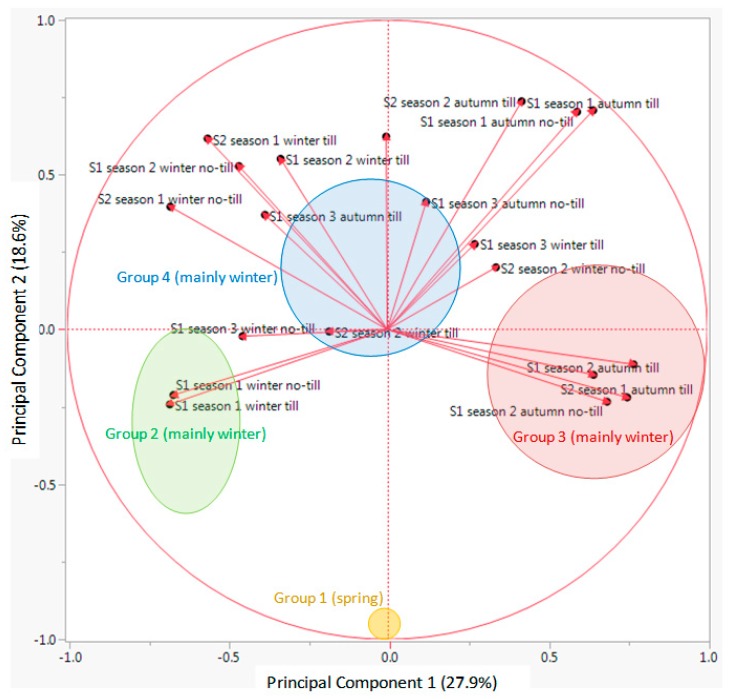
Biplot representation of the variables (vectors) used in a PCA of the percentage of emergence of the 30 RAP in autumn, winter or spring in two different trials under two tillage regimes. In each variable, S1 refers to the trial started in 2012, S2 for the trial started in 2013; season 1 for 2012/2013, season 2 for 2013/2014, and season 3 for 2014/2015; autumn, winter or spring, the period from which the emergence is provided; there were two burial conditions: 1 cm burial depth without soil disturbance (referred as no-till in the vectors) and 1–10 cm depth with annual autumnal soil disturbance (referred as till in the vectors). Vectors are shown as the eigenvectors of the covariance matrix scaled by the square root of the corresponding eigenvalue and shifted so their tails are at the mean. Relative position of the four groups of RAP by means of K-means clustering (see Table 2 and Figure 5) shown in circles (where 50% of observations would be found) in different colors.

Looking at the results from the K-means clustering (together with the CHC) and from the PCA, particularly the biplot for the vectors (Figure 5), the nature of each group was clear regarding its emergence pattern. The Y axis (PC 2) distributed the RAP species from those almost only germinating in spring (group 1, only with *G. ladanum*), from the rest. The X axis (PC 1) distributed the species from those with emergence concentrated in winter and also important emergence in spring (group 2, *A. flammea*, *H. pendulum*, *Legousia hybrida*, *N. gallica*, and *T. latifolia*), from those emerging mainly in autumn, with some winter emergence (group 3, mostly *Cariophyllaceae*, and other species) and group 4 (mainly *Ranunculaceae*, and some *Apiaceae* among others) that emerged both in autumn and winter (Table 2).

Looking at the output from PCA biplot for the vectors of each data set (Figure 5), it seems that the first PC (X axis) distributed the 30 RAP species depending on an emergence pattern more concentrated in autumn or more in winter: species located on the right side of this axis had important levels of emergence in autumn, while species located on the left side had important emergence flushes in winter. On the other hand, the second PC (Y axis) distributed species in relation to emergence in spring or the rest of the season. Species with most emergence in spring were located in the bottom part of the biplot (Figure 4), while species with more emergence in autumn and/or winter were located in the upper part of the graph. Finally, considering the distribution of the data vectors in the PCA (Figure 5), no-till or till, burial conditions did not seem to have major effects on relative emergence patterns of the 30 RAP studied

## 3. Discussion

According to the results presented in this research, the 30 RAP species studied could be divided into four distinct groups according to their relative emergence levels during the germinating season from autumn to spring. Looking at the results from the multivariate analyses, the nature of each group was clear regarding its emergence pattern: species almost only germinating in spring, the species with emergence concentrated in winter and also important emergence in spring, those emerging mainly in autumn with some winter emergence, and finally those that emerged both in autumn and winter. Interestingly, similar results were obtained in a previous study where late germinating species (*Galeopsis* sp.) were clearly separated from the rest with similar multivariate analysis [27].

The data used in this research had two soil burial conditions: 1 cm depth without soil disturbance (no-till), and 1–10 cm depth with annual autumnal soil disturbance (till). Even though these burial conditions could delay or advance emergence, there were no clear effects on the emergence patterns; for a given species one season in one trial (S1 or S2) burial could advance or delay emergence (Figure A1, Figure A2, Figure A3, Figure A4 and Figure A5). Seemingly, the response of emergence to tillage was species-specific and more dependent on environmental factors such as rainfall [28]. On the other hand, tillage promoted the total seedling emergence of these RAP in the same study [5] and, thus, tillage remains a relevant strategy to build RAP soil seed reserves and a viable technique for management and conservation aims [29]. Conversely, the growing no-tillage cultivation could have opposite and detrimental effects in arable farming on RAP.

Weed emergence patterns are primarily influenced by temperature and secondarily by soil moisture, but also reflect species-specific temperature and moisture germination requirements [28]. Emergence patterns of all the 30 RAP the second season in trial S1 (2013/2014 in Figure 1) that were more than one year old and buried seeds, were very similar to the first season in trial S2 (also 2013/2014 in Figure 2), that is, seeds collected and buried that year. Almost all species (24 out of 30) had emergence concentrated (≥60%) in winter and spring that season irrespective of the group to which they belonged. Winter in 2013/2014 was the wettest and also cold (Table 1). Apparently, temperature and rainfall seemed to be more important to explain the variability in emergence patterns than other factors such as seed age and time of burial, or even the species. This strong effect of seasonal weather variations on the total cumulative emergence in these experiments was already assessed and reported in previous research [5].

On a species basis, this research corroborated that those of genus *Galeopsis* are obligate spring-germinating species and require higher temperatures [27,30]. Emergence has been studied in depth for some of the species, and for example, *Thlaspi arvense*, *C. microcarpa*, and *N. paniculata* mainly emerged in autumn and winter too [31,32,33]. Also, for species belonging to *Papaveraceae*, main flushes were detected in autumn and winter, like *Papaver argemone*, which showed cooler germination requirements according to emergence distribution compared to the other species in this family [25,34]. *Consolida* species can germinate mainly in autumn–winter, while *Delphinium* species and *N. gallica* also germinate in spring [7]. The clustering in this research corroborated this pattern for *Consolida* species and *N. gallica*, but not for *Delphinium* species (Table 2).

### Potential Implications for Management

Some management recommendations can be derived to help optimize survival in the studied RAP species in arable landscapes. Aspects of soil preparation for fields, such as the timing of cultivation and seeding, can increase the occurrence of RAP [25]. The first important decision concerns the optimal time of sowing, i.e., in relation to the sowing time of the crop or the emergence peak of competitive weeds. Results indicate that species belonging to group 3 (mainly autumn, Table 2) could successfully be reintroduced only with an early sowing in autumn, both in cropped fields or non-cropped field margins. On the other hand, delayed sowing of species from groups 2 (mainly winter) and 4 (autumn-winter) in Table 2 could be another management option for restoring or managing their populations. Target species of these two groups, particularly those of group 2, seemed to be well adapted to sowing delays in late autumn or early winter. Moreover, for conservation purposes, cultivation and sowing of these species would be more promising during the cold part of the year. Flexibility in the crop sowing date would help in diversifying crop rotations, for example, with less competitive crops compared to cereals [35]. Additionally, later crop sowings could reduce competition between RAP and dominant noxious weeds, which can have an emergence peak in early autumn [21]. Avoiding competition with dominant species can be crucial for the success of conservation efforts [36].

The next step will be to develop common emergence models for these four groups of RAP species established in this study according to emergence patterns. These common emergence models will aid in establishing more precise conservation and management recommendations for these species under threat across Europe. This is important as this research showed that environmental factors, mainly temperature and rainfall, play a key role, given that every season emergence patterns were similar in S1 and S2 trials. Moreover, these models will also help in understanding temperature, soil water, light, and tillage requirements for emergence for these groups of RAP. If developed, this information about emergence patterns will be critical, for example, in deciding when and which type of herbicides (contact or residual) can be applied. Chemical management can be important for controlling harmful weeds, while conserving RAP species [22]. Another step may be to purposefully test whether recommendations given in this study really work. A few distinctly contrasting species for each group could be chosen, sown at optimal and suboptimal seasonal timings, and then their fitness, particularly with respect to total seed production, measured. These results are based on a one-field study and therefore an expansive verification would be necessary for e.g., a Spain- or Europe-wide extrapolation.

## 4. Materials and Methods

### 4.1. Plant Material

Seeds of 30 RAP species were harvested from winter cereal fields in Spain from late June through August in two consecutive years (2012 and 2013) as described in Torra et al. [5]. Collection sites were located in two previously surveyed areas that host high arable plant diversity: the provinces of Teruel and Lleida [5]. The details (location, coordinates and collection dates) of the 30 species studied, belonging to eight different botanical families, are available at Torra et al. [5]. All species studied are naturally occurring as rare in the region [7,17,18,26,37].

Seeds were air-dried for one week after collection, cleaned and then stored in dry conditions with silica gel at room temperature (around 21 °C) until experiments started. Seeds selected for the trials were counted either manually or with a seed counter (Contador E 230 V; Pfeuffer GmbH, Kitzingen, Germany). Poor-quality seeds were discarded based on seed firmness and the presence of mold, thus, only high-quality seeds were used [5].

### 4.2. Experimental Design

The seeds of the 30 RAP species were sown in a fallow part of a winter cereal field, with a soil texture of 47.4% sand, 18.4% clay, and 34.2% silt, pH 8.2 and 2.9% of organic matter, located in Almenar (Lleida, Spain; 41°46′36″ N, and 00°32′07″ E). The experiment was duplicated in two consecutive trials, the first initiated on 28 August 2012 (assessment started in season 2012/2013 or trial S1), and the second on 28 August 2013 (assessment started in season 2013/2014 or trial S2). These dates allowed seeds to experience natural temperature and humidity fluctuations for about two months until typical cereal sowing dates in autumn, which corresponds to the beginning of the winter cereal season.

RAP seeds were sown considering two tillage treatments: seeds sown at 1 cm depth; and seeds sown and homogeneously distributed between 1 and 10 cm depth. The 1 cm depth simulated no-till conditions where seeds are maintained at or near the soil surface covered by a thin layer of soil; the contrasting treatment simulated soil tillage (e.g., disking) where seeds are evenly mixed between the 1 to 10 cm soil depths using hoes. The number of seeds sown in each plot varied according to availability among species, ranging between 100 and 1000 seeds. The exact number of sown seeds, emerged plants and cumulative emergences can be found in Torra et al. [5].

**Figure 6:**
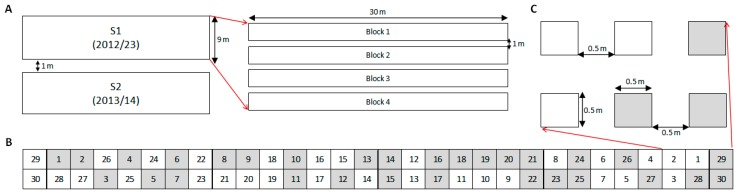
Design of the experiment duplicated into two trials (S1 and S2), where 30 RAP were sown to monitor emergence patterns during three consecutive seasons. (**A**) Relative locations and dimensions of S1 and S2, season of establishment in parenthesis, and blocks distribution of one of the trials as example. (**B**) Randomization of the 30 RAP (each number represents a species) for a block, where two burial conditions are shown: 1 cm burial depth without soil disturbance (in white) and 1–10 cm depth with annual autumnal soil disturbance (in grey). (**C**) Details of individual plots arrangement and size.

Each experiment (S1 and S2) was arranged in a completely randomized block design with four replications. RAP species were sown in single plots: four plots in no-till and four plots with soil disturbance. Therefore, 240 plots were established in each trial and sown with individual species per tillage treatment. Plot size was 0.5 by 0.5 m, buffered with 0.5 m corridors, and 1 m alleys between blocks (Figure 6). On 2 November 2012, 28 October 2013 and 16 October 2014, tilled plots were hoed with hand-tools to simulate a tillage operation, which are usual timings for tillage and sowing operations in the area. Emergence was monitored weekly with destructive counts from soil disturbance until May when emergence ceased. S1 (started in 2012) was monitored over three consecutive seasons, 2012–2015, while S2 (started in 2013) was monitored over two consecutive seasons, 2013–2015.

The main weed species present in the experimental field were *Papaver rhoeas*, *Lolium rigidum*, *Sisymbrium irio*, and *S. crassifolium*, and to a lesser extent some Asteraceae (*Anacyclus clavatus*, *Cirsium arvense*, and *Conyza* sp.). Weed densities were very low (<5 plants m^−2^) because the field was ploughed twice per year to keep it as fallow for endangered bird species, since it is located in a protected IBA (Important Bird Area) area. The RAP species studied were not present in the field.

### 4.3. Data

To reduce the amount and simplify data handling, the percentage of emergence was divided into and summarized in three periods: Autumn (November through December), Winter (January through February), and Spring (March through April). For each of these three periods relative emergence of each species was calculated both with and without soil disturbance. This was done for the following series of data: (1) first season cumulative emergence after burial was calculated as percentage of sown seeds for S1 in season 2012/2013, and for S2 in season 2013/2014; (2) second season cumulative emergence after one year of burial was calculated for S1 for 2013/2014, and for S2 in 2014/2015, subtracting previous cumulative emergence during the first season; and (3) third season cumulative emergence after two years of burial was calculated for S1 for 2014/2015, subtracting previous cumulative emergence from initially sown seeds. Daily rainfall and temperature were obtained from a meteorological station situated 4 km away from the experimental site.

### 4.4. Statistical Analysis

Data, namely that is relative emergence in Autumn, Winter and Spring for the series in trials S1 and S2 during three consecutive seasons (only for S1) under two tillage regimes, were analyzed using multivariate techniques. Since relative emergence each season was complementary (always summed to 1) and linearly correlated, relative emergence in spring was not included in the analyses. This same raw data, 30 rows (one per species) and 20 columns, was used in all the analyses detailed below. In order to detect groups of RAP species with similar emergence patterns inside each cluster, but clearly different from any other clusters, we used K-means clustering algorithms [38]. The K-means is an iterative, data-partitioning algorithm and requires as input a matrix of points in n dimensions and a matrix of K initial cluster exemplars in n dimensions (determined using the Euclidean distance between point and cluster). The general procedure is to search for a K-partition with locally optimal within-cluster sum of squares by moving points from one cluster to another [38]. In order to determine the optimal number of clusters, we applied the Calinski-Harabasz criterion (CHC) to the K-means clustering [29]. CHC minimizes the within-group sum of squares and maximizes the between-group sum of squares. The highest CHC value corresponds to the optimal set (of most compact clusters). The optimal set can be recognized by a peak or at least an abrupt elbow on the linear plot of CHC values. By contrast, if the line is horizontal, smooth, ascending or descending, then it is not possible to find an optimal set [39]. Besides, in order to visualize in a two-dimensional scale the RAP classification obtained according to the K-means clustering and CHC analysis, a Principal Component Analysis (PCA) was performed on the data. The first two Principal Components (PC) from the PCA were used to graphically represent the distribution of the clusters obtained. K-Means Cluster Analyses were performed using the “apcluster” software package implemented in the free statistical application, R [40], while the PCA was done with JMPro14 software (SAS Campus Drive, Cay, NC, USA).

## Figures and Tables

**Table 1:** Monthly mean temperature (°C) and cumulative rainfall (mm) from September to April) during three observation seasons.

			Seasons			
	2012/2013		2013/2014		2014/2015	
Month	Mean Temperature	Rainfall	Mean Temperature	Rainfall	Mean Temperature	Rainfall
September	20.0	49	7.7	5	10.8	146
October	15.7	92	5.0	6	6.7	17
November	9.5	30	3.1	56	5.9	123
December	5.9	7	0.1	12	0.7	11
January	4.7	31	2	37	0.2	12
February	5.8	8	1.4	14	0.1	13
March	9.4	68	2.3	13	3.0	45
April	11.9	78	5.0	46	3.4	8

**Table 2:** Groups defined according to the K-means cluster analysis. Letters in parenthesis represent the code used for graphical representation of the K-means clustering on a PCA biplot (Figure 5).

Groups	Emergence Patterns	RAP Species
Group 1	spring	*Galeopsis ladanum* (Gal lad)
Group 2	mainly winter	*Adonis flammea* (Ado fla), *Hypecoum pendulum* (Hyp pen), *Legousia hybrida* (Leg hyb), *Nigella gallica* (Nig gal), *Turgenia latifolia* (Tur lat)
Group 3	mainly autumn	*Agrostemma githago* (Agro git), *Androsace maxima* (And max), *Biscutella auriculata* (Bis aur), *Camelina microcarpa* (Cam mic), *Cerastium perfoliatum* (Cer per), *Neslia paniculata* (Nes pan), *Papaver dubium* (Pap dub), *Silene conoidea* (Sil con)
Group 4	autumn-winter	*Adonis aestivalis* (Gal lad), *Asperula arvensis* (Asp arv), *Bifora radians* (Bif rad), *Bupleurum rotundifolium* (Bup rot), *Conringia orientalis* (Con rin), *Consolida orientalis* (Con ori), *Consolida pubescens* (Con pub), *Delphinium gracile* (Del gra), *Delphinium halteratum* (Del hal), *Iberis amara* (Ibe ama), *Lathyrus aphaca* (Lat aph), *Papaver argemone* (Pap arg), *Ranunculus arvensis* (Ran arv), *Roemeria hybrida* (Roe hyb), *Thlaspi arvense* (Thl arv), *Vaccaria hispanica* (Vac his)
